# Impact of Carbon Nanomaterials on the Antioxidant System of Tomato Seedlings

**DOI:** 10.3390/ijms20235858

**Published:** 2019-11-22

**Authors:** Yolanda González-García, Elsy Rubisela López-Vargas, Gregorio Cadenas-Pliego, Adalberto Benavides-Mendoza, Susana González-Morales, Armando Robledo-Olivo, Ángel Gabriel Alpuche-Solís, Antonio Juárez-Maldonado

**Affiliations:** 1 Doctorado en Ciencias en Agricultura Protegida, Universidad Autónoma Agraria Antonio Narro, Saltillo, Coahuila 25315, Mexico; yolanda_glezg@hotmail.com (Y.G.-G.); lopez2690vargas@gmail.com (E.R.L.-V.); 2 Centro de Investigación en Química Aplicada, Saltillo, Coahuila 25294, Mexico; gregorio.cadenas@ciqa.edu.mx; 3 Departamento de Horticultura, Universidad Autónoma Agraria Antonio Narro, Saltillo, Coahuila 25315, Mexico; adalberto.benavides@uaaan.edu.mx; 4 CONACyT- Departamento de Horticultura, Universidad Autónoma Agraria Antonio Narro, Saltillo, Coahuila 25315, Mexico; qfb_sgm@hotmail.com; 5 Departamento de Alimentos, Universidad Autónoma Agraria Antonio Narro, Saltillo, Coahuila 25315, Mexico; armando.robledo@uaaan.edu.mx; 6 Instituto Potosino de Investigación Científica y Tecnológica, San Luis Potosí, San Luis Potosí 78216, Mexico; alpuche@ipicyt.edu.mx; 7 Departamento de Botánica, Universidad Autónoma Agraria Antonio Narro, Saltillo, Coahuila 25315, Mexico

**Keywords:** carbon nanotubes, graphene, antioxidant compounds, enzymatic activity, oxidative stress

## Abstract

Tomato is one of the most economically important vegetables worldwide and is constantly threatened by various biotic and abiotic stress factors reducing the quality and quantity in the production of this crop. As an alternative to mitigate stress in plants, carbon nanomaterials (CNMs) have been used in agricultural areas. Therefore, the objective of the present work was to evaluate the antioxidant responses of tomato seedlings to the application via foliar and drench of carbon nanotubes (CNTs) and graphene (GP). Different doses (10, 50, 100, 250, 500, and 1000 mg L^−1^) and a control were evaluated. The results showed that the fresh and dry root weight increased with the application of CNMs. Regarding the antioxidant responses of tomato seedlings, the application of CNMs increased the content of phenols, flavonoids, ascorbic acid, glutathione, photosynthetic pigments, activity of the enzyme’s ascorbate peroxidase, glutathione peroxidase, catalase, and phenylalanine ammonia lyase as well as the content of proteins. Therefore, the use of carbon-based nanomaterials could be a good alternative to induce tolerance to different stress in tomato crop.

## 1. Introduction

Advances in nanotechnology have provided new materials with various applications. Proof of this are the so-called carbon nanomaterials (CNMs) that have been used in various areas including agriculture [1]. These CNMs have the characteristic that they can be easily absorbed by plant cells causing positive impacts on plant growth and development [2]. Among the application highlights are the high potential for the elimination of pesticides in water [3], ability to remove heavy metals from soil and water [4], and antifungal and bactericidal effects. In agriculture, they have been applied to stimulate seed germination [1], and they also act as growth regulators [5] and stimulate the antioxidant activity of plants [2].

Among the most studied CNMs in agriculture are graphene, carbon nanotubes, graphite, and fullerene [5]. Graphene (GP) is a nanostructure composed of a single layer of carbon atoms densely packed in a benzene ring structure [6] with thermodynamically favorable configurations of sp^3^ to sp^2^, depending on the heat of the formation and the pressure conditions [7]. It has a carbon–carbon bond length of approximately 0.142 nm and the diameter of the graphene layer is approximately 0.35 nm [8]. This gives it unique properties such as a two-dimensional flat structure, large surface area, chemical and mechanical stability, excellent conductivity, good biocompatibility, and ease of functionalization [9,10]. On the other hand, carbon nanotubes (CNTs) are rolled laminar structures of hybridized carbon atoms (sp^2^) arranged hexagonally, forming hollow cylindrical tubes [11]. Its dimensions are a few nanometers in diameter (up to approximately 100 nm) and lengths in the micrometer range [12]. Depending on the number of graphene layers, they are classified as single-walled CNTs (SWCNTs) or multiple-walled CNTs (MWCNTs) [12]. Carbon nanotubes are well assembled and have a good surface area, high strength, and excellent chemical and thermal properties [11]. It has been shown that both graphene and carbon nanotubes are biocompatible materials [9,13]; thus, they can be applied in plants.

It is well known that the toxicity of CNMs depends mainly on the dose applied, although the exposure time and the plant species used also has an influence [14]. It has been shown that doses of 500–2000 mg L^−1^ of graphene in a nutritive solution for 20 days inhibits growth and increases the concentration of reactive oxygen species (ROS) in seedlings of cabbage (*Brassica oleracea*), tomato (*Solanum lycopersicum*), and red spinach (*Spinacia oleracea*); however, no toxic effect on the seedlings of lettuce (*Lactuca sativa*) was observed [15]. In the case of CNTs, a dose of 1000–2000 m L^−1^ in nutritive solution for 15 days affected root growth and shoots in red spinach (*S. oleracea*), lettuce (*L. sativa*), and cucumber (*Cucumis sativus*) but had no toxic effect on chili pepper (*Capsicum annuum*), lady’s finger (*Sedum pachyphyllum*), and soybeans (*Glycine max*) [16]. In seedlings of *Hyoscyamus niger* treated with CNTs at concentrations of 400 and 800 mg L^−1^, a decrease in germination and yield was observed; however, at low doses (50 and 100 mg L^−1^), they presented the opposite effect, since the germination and vigor of the plants increased [17].

In addition to its economic relevance, tomato has been identified as a food of great interest due to the fact of its high content of bioactive compounds [18]. Tomato, like all crop plants, are subject to various types of stress, both biotic and abiotic [19]. Therefore, the application of nanomaterials as biostimulants is interesting [20] as an alternative to increase the yield, quality, productivity, and defense of plants [21]. However, to achieve this, an understanding of how CNMs act is required; thus, the present research aimed to evaluate the antioxidant responses and growth of tomato seedlings treated with CNMs—carbon nanotubes and graphene—through two routes of application.

## 2. Results

### 2.1. Crop Growth

**Figure 1:**
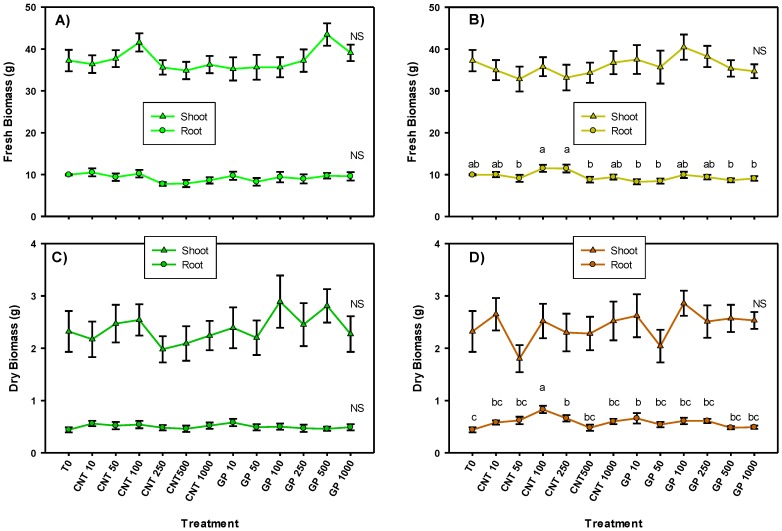
Shoot and root biomass of tomato seedlings with carbon nanomaterials applied via foliar (**A**,**C**) and drench (**B**,**D**). T0: control; CNTs: carbon nanotubes; GP: graphene; DW: dry weight; 10, 50, 100, 250, and 500 represent the mg L^−1^ applied of each carbon nanomaterial. Different letters indicate significant differences among treatments according to Duncan (*α* = 0.05). *n* = 5 ± standard error.

The results obtained showed that when the CNMs were applied via foliar, there was no impact on the shoot and root biomasses, indicating that there was no toxicity due to the presence of any of the doses used (Figure 1). When the application was done via drench, only significant differences were observed among treatments in the root biomass. In the fresh root biomass, there were no differences compared with the control. While in the dry root biomass, 100 mg L^−1^ of CNT was the best treatment with 87% more biomass than the control. Also 250 mg L^−1^ of CNT and 10 mg L^−1^ of GP generated more root biomass than the control by 47%.

### 2.2. Photosynthetic Pigments

**Figure 2:**
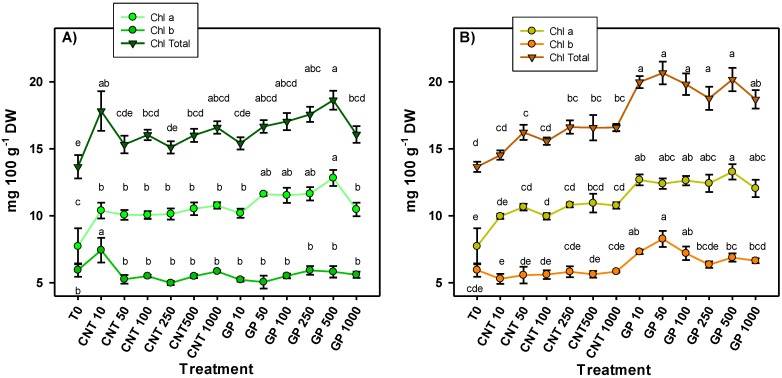
Photosynthetic pigments in tomato seedlings treated with carbon nanomaterials applied via foliar (**A**) and drench (**B**). Chl a: chlorophyll a; Chl b: chlorophyll b; Chl Total: sum of chlorophyll a + chlorophyll b; T0: control; CNTs: carbon nanotubes; GP: graphene; DW: dry weight; 10, 50, 100, 250, and 500 represent the mg L^−1^ applied of each carbon nanomaterial. Different letters indicate significant differences among treatments according to Duncan (*α* = 0.05). *n* = 5 ± standard error.

The chlorophyll content in the leaves of tomato seedlings treated with graphene and carbon nanotubes showed significant differences among treatments (Figure 2). The results were consistent between both routes of application, since all treatments applied to CNTs and GP increased the chlorophyll a content. The application of GP at 500 mg L^−1^ induced the best response being 66% more than the control in the foliar application and 72% more in the application via drench. In addition, there was a clear trend that treatments with graphene application induced a higher chlorophyll content compared to the application of CNTs, regardless of the route of application. Derived from the increase observed in chlorophyll a, an increase in total chlorophyll was also observed. On the other hand, chlorophyll b increased with the application of graphene via drench at 10, 50, and 100 mg L^−1^ by 23%, 39%, and 21%, respectively, compared to the control. In foliar application, only the 10 mg L^−1^ of CNT treatment showed an increase in chlorophyll b by 25% compared to the control.

### 2.3. Non-Enzymatic Antioxidant Compounds

**Figure 3:**
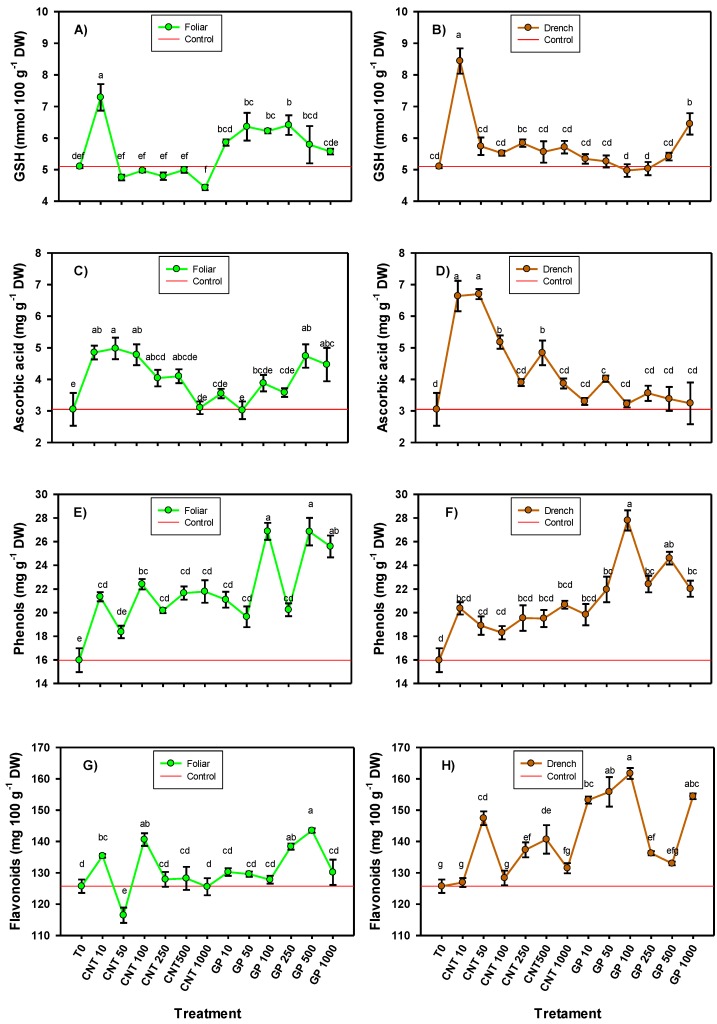
Glutathione (**A**,**B**), ascorbic acid (**C**,**D**), phenols (**E**,**F**), and flavonoids (**G**,**H**) in tomato seedlings with the application of carbon nanomaterials. T0: control; CNTs: carbon nanotubes; GP: graphene; DW: dry weight; 10, 50, 100, 250, and 500 represent the mg L^−1^ applied of each carbon nanomaterial. Different letters indicate significant differences among treatments according to Duncan (*α* = 0.05). *n* = 5 ± standard error.

The application of CNMs modified the content of the non-enzymatic antioxidant compounds (glutathione, vitamin C, phenols, and flavonoids), generally in a positive way (Figure 3). The glutathione content presented the greatest increase with the application of CNTs at 10 mg L^−1^, both in foliar application (42.94%) and in drench application (65.49%). However, when graphene was applied, the behavior was different depending on the route of application. By foliar route, the doses of 50, 100, and 250 mg L^−1^ increased the glutathione content compared to the control by 25.7%, 24.7%, and 22%, respectively; while in the application via drench, only the dose of 1000 mg L^−1^ induced an increase (26.47%) compared with the control.

The ascorbic acid content was positively modified by the application of CNMs regardless of the route of application, especially with CNTs (Figure 3). The doses of 10, 50, and 100 mg L^−1^ of CNT generated the highest content of this compound in foliar application, up to 59%, 63%, and 57%, respectively, compared with the control. When applied via drench, doses of 10, 50, 100, and 500 mg L^−1^ of CNT induced an increase in ascorbic acid compared with the control by 118%, 120%, 70%, and 59%, respectively. These results indicate that the application of CNTs via drench induced a better response in the content of ascorbic acid compared to the application via foliar. In contrast, the application of graphene via foliar induced a better response than when applied via drench. Doses of 500 mg L^−1^ followed by 1000 mg L^−1^ applied via foliar induced the highest concentration compared to the control (55% and 46% more, respectively), while, via drench, only the graphene at 50 mg L^−1^ induced a positive response compared with the control (31.8%).

The phenols content consistently increased with the application of both CNTs and graphene for both routes of application (Figure 3). Likewise, it was observed that graphene induced better responses than CNTs, mainly the doses of 100 and 500 mg L^−1^ of GP. When applied by foliar route, both doses of GP increased the phenols content by 68% compared with the control, and, when they were applied via drench, there was an increase of 74% with 100 mg L^−1^ and 54% with 500 mg L^−1^.

In the case of flavonoids, the best response was observed when the application was done via drench and especially with graphene (Figure 3). The application of 100 mg L^−1^ of graphene via drench induced the greatest number of flavonoids, 28.6% more than the control; although, the doses of 10, 50, 250 and 1000 mg L^−1^ were also better than the control. As for the CNTs applied via drench, the dose of 50 mg L^−1^ showed an increase of 17.3%; in addition, the doses of 250 and 500 mg L^−1^ also showed an increase of 9.3% and 11.9%, respectively, compared with the control. When the application was done via foliar, the doses of 500 and 250 mg L^−1^ of graphene induced the greater accumulation of flavonoids, being 14% and 10% more than the control, respectively. In the case of the CNTs applied via foliar, an increase of 11.8% and 7.7% was observed with the doses of 100 and 10 mg L^−1^, respectively.

**Figure 4:**
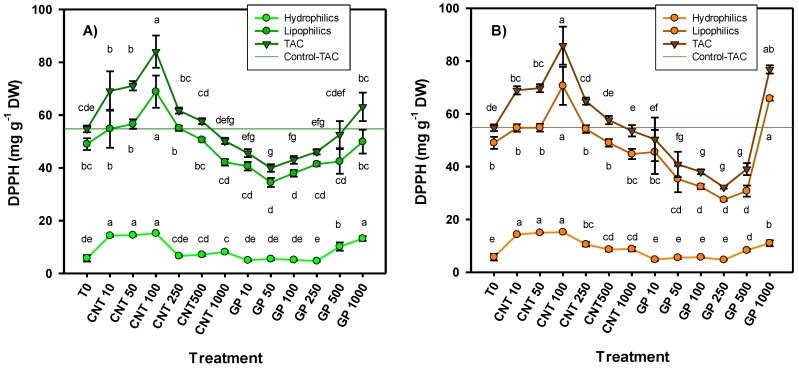
Antioxidant capacity in tomato seedlings treated with carbon nanomaterials applied via foliar (**A**) and drench (**B**). TAC: total antioxidant capacity. T0: control; CNTs: carbon nanotubes; GP: graphene; DW: dry weight; 10, 50, 100, 250, and 500 represent the mg L^−1^ applied of each carbon nanomaterial. Different letters indicate significant differences among treatments according to Duncan (*α* = 0.05). *n* = 5 ± standard error.

The antioxidant capacity determined by the DPPH (2,2-diphenyl-1-picrylhydrazyl) radical was consistent for both routes of application, since, in both cases, the treatment of CNT at 100 mg L^−1^ generated the best response, increasing by 53.5% in foliar application and 56.8% in application via drench compared with the control (Figure 4). Also, doses of 10 and 50 mg L^−1^ induced a favorable response in antioxidant capacity. As for graphene, the highest dose (1000 mg L^−1^) was the one that induced the best result in antioxidant capacity for both routes of application; however, only in the drench route application was there a significant difference compared to the control (40% more).

### 2.4. Protein Content and Enzymatic Activity

**Figure 5:**
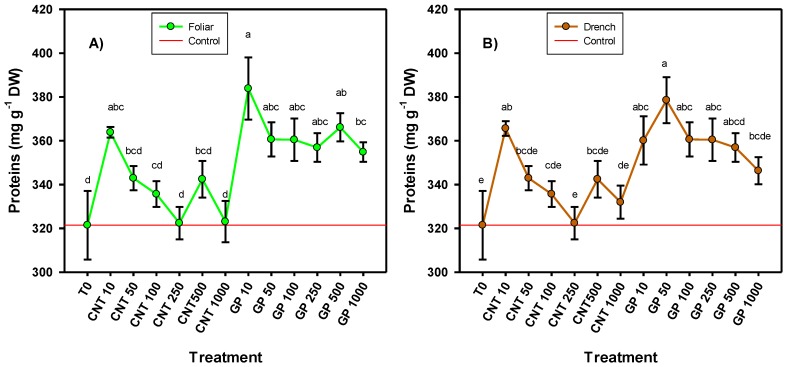
Protein content in tomato seedlings treated with an application of carbon nanomaterials applied via foliar (**A**) and drench (**B**). T0: control; CNTs: carbon nanotubes; GP: graphene; DW: dry weight; 10, 50, 100, 250, and 500 represent the mg L^−1^ applied of each carbon nanomaterial. Different letters indicate significant differences among treatments according to Duncan (*α* = 0.05). *n* = 5 ± standard error.

The protein content was positively affected by the application of CNMs. In both routes of application, very similar results were observed, where graphene was the one that induced the best response (Figure 5). When CNTs were applied, only the lowest dose (10 mg L^−1^) induced an increase compared to the control in both the foliar (19.4%) and the drench routes (13.7%). When graphene was applied, virtually all doses applied induced higher protein content. In the foliar application, 10 mg L^−1^ of graphene induced the greatest amount of proteins, 19.4% more than the control, while in application via drench, 50 mg L^−1^ graphene induced 17.8% more proteins than the control.

**Figure 6:**
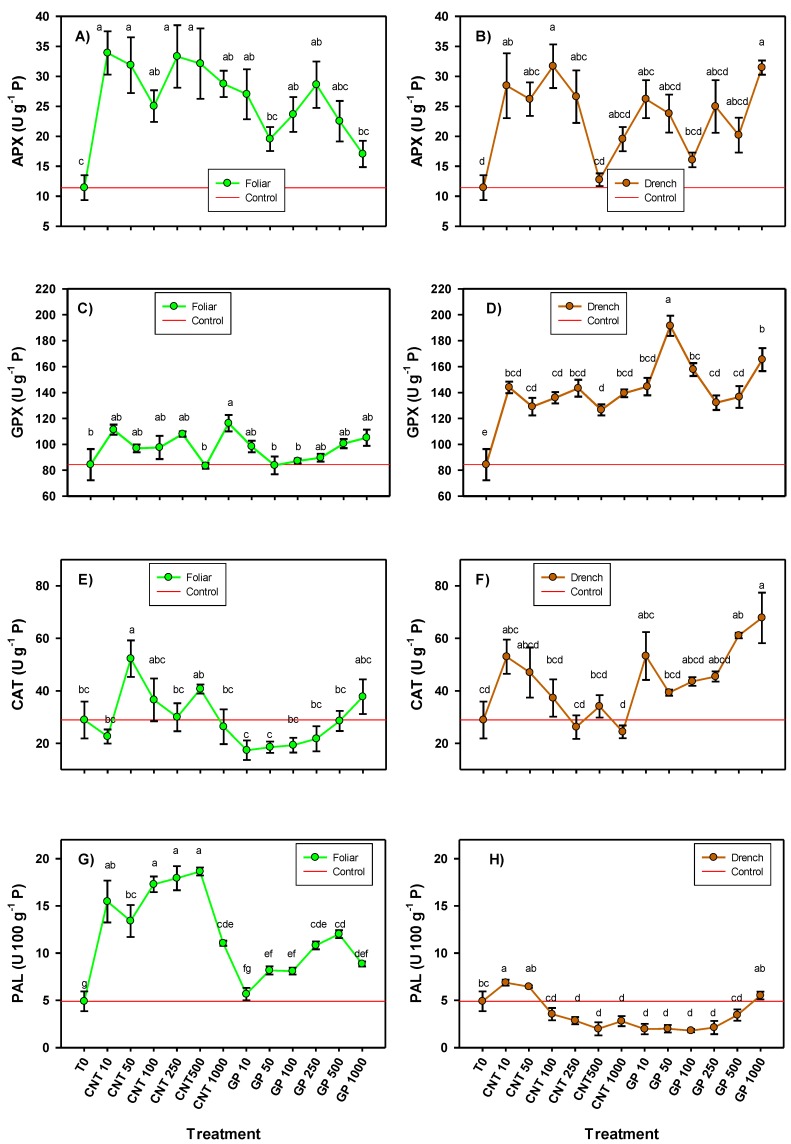
Enzymatic activity (ascorbate peroxidase (**A**,**B**), glutathione peroxidase (**C**,**D**), catalase (**E**,**F**), and phenylalanine ammonia lyase (**G**,**H**) in tomato seedlings treated with carbon nanomaterials applied via foliar and drench. T0: control; CNTs: carbon nanotubes; GP: graphene; P: proteins; 10, 50, 100, 250, and 500 represent the mg L^−1^ applied of each carbon nanomaterial. Different letters indicate significant differences among treatments according to Duncan (*α* = 0.05). *n* = 5 ± standard error.

The activity of all the enzymes evaluated was modified by the application of the CNMs, although some enzymes also presented a different response depending on the route of application (Figure 6). Foliar application of all CNT doses induced a greater activity of the APX enzyme compared with the control (119–196%), while, with graphene, only the doses of 10, 100, and 250 mg L^−1^ induced greater APX activity in comparison with the control in a range of 107–150%. When the application was via drench, doses of 10, 50, 100, and 250 mg L^−1^ of CNT increased APX activity compared to the control in a range of 129–177%; while graphene at 1000 mg L^−1^ increased APX activity by 175%, followed by 10 mg L^−1^ with an increase of 129%.

The GPX activity presented very different results when the CNMs were applied via foliar or via drench (Figure 6). In foliar application, only the 1000 mg L^−1^ dose of CNT induced an increase in GPX activity, 39.6% more than the control. In application via drench, all CNMs treatments increased GPX activity, especially with the application of graphene. The CNT increased the activity of GPX in a range of 50–70% compared to the control, while graphene treatments induced an increase in a range of 57–127% with the dose of 50 mg L^−1^ which generated the best response.

The catalase activity presented different changes according to the route of application of the CNMs (Figure 6). In foliar application, 50 mg L^−1^ of CNT was the only treatment that induced greater activity of this enzyme with an increase of 81% compared with the control. In application via drench, doses of 1000 and 500 mg L^−1^ of graphene induced an increase of 135% and 111%, respectively.

The activity of the phenylalanine ammonia lyase (PAL) enzyme was also affected differently depending on the route of application of the CNMs, since, in foliar application, with the exception of one treatment, the rest induced greater activity compared with the control, while via drench, only the application of 10 mg L^−1^ of CNT induced an increase of 40% compared with the control. In the foliar application, differences were also observed among the types of CNMs used, since the application of CNTs generated a better response than graphene in PAL activity. Doses of 100, 250, and 500 mg L^−1^ induced the greatest increase in PAL activity, 254%, 267%, and 281% more than the control, respectively. With graphene, the best result was obtained with 500 and 250 mg L^−1^ which increased the PAL activity up to 146% and 121%, respectively, compared with the control.

## 3. Discussion

The increase in root biomass may be related to the fact that CNMs have the ability to bind and penetrate the root surface which improves the capillary action of water and nutrient absorption [22]. In addition, they act as elicitors in the regulation of plant growth [5], since they activate the biosynthesis of indole acetic acid and abscisic acid [23]. They also promote the expression of marker genes of cell division (*CycB*) and elongation of the cell wall (*NtLRX1*) [24] that directly influence growth. Additionally, they increase the activity of the enzymes superoxide dismutase (SOD), guaiacol peroxidase (POD), catalase (CAT), and ascorbate peroxidase (APX), resulting in the accumulation of proteins in the roots [25] which can increase disease resistance [5] and, ultimately, contribute to healthier and more vigorous roots. Similar results have been reported by various authors. Tripathi et al. [22] demonstrated a significant increase in the growth rate of chickpea (*Cicer arietinum*) plant roots with the application of CNTs to 100 mg L^−1^ in seeds. Joshi et al. [26] reported an increase in the number and length of radicle in wheat (*Triticum aestivum*) plants with CNT application. In contrast, Begum et al. [15], reported that the application of graphene in doses of 500, 1000, and 2000 mg L^−1^ significantly affected the size and weight of the roots in cabbage (*Brassica oleracea*), tomato (*Solanum lycopersicum*), and red spinach (*Spinacia oleracea*) crops. This indicates that the responses may be different depending on the type of CNMs used, the dose applied as well as the route of application.

As an essential cofactor of photosystem I and II, chlorophylls play a fundamental role in photosynthesis, since they are responsible for both the absorption of visible light and its photochemical conversion within the cell [27]. Chlorophyll a is in a higher concentration than chlorophyll b, commonly 3:1 [28], so chlorophyll a is more susceptible to being affected by environmental factors as CNMs. Both carbon nanotubes and graphene have the ability to penetrate the cells of the epidermis and transport via endocytosis to cell walls, mitochondria, and chloroplasts [29]. In spinach plants (*S*. *oleracea*), the application of carbon nanotubes increased electron flow and photosynthetic activity due to the stimulating action on light absorption caused by the penetration of CNTs into chloroplast membranes [30]. Likewise, Giraldo et al. [31] showed that the application of carbon nanomaterials in *S*. *oleracea* increased the number and size of chloroplasts, and, as a result, there was an increase in chlorophylls and photosynthetic activity. Begum et al. [16] reported an increase in chlorophyll a and b content with the addition of carbon nanotubes in a wild carrot crop (*Daucus carota*). Siddiqui et al. [32] also reported an increase in carrot chlorophyll content with the application of graphene oxide at 10 mg L^−1^. The results obtained in this work are consistent with those reported in the literature which shows that the application of CNMs induces favorable responses in plant photosynthetic pigments which can potentially increase photosynthetic capacity.

The antioxidant defense system of plants works from a set of enzymatic (APX, GPX, CAT, SOD, etc.) and non-enzymatic (glutathione, ascorbic acid, phenols, etc.) antioxidant compounds which participate directly or indirectly in the control of reactive oxygen species. Glutathione (GSH) is a molecule composed of three amino acids, L-cysteine, L-glutamic acid, and glycine, and acts as an antioxidant [33]. In plants, GSH plays a very important role, since together with ascorbic acid it is part of the ascorbate–glutathione cycle [34]. In addition, it improves osmoregulation, the efficiency of water and nutrient use, and photosynthetic performance [35]. On the other hand, ascorbic acid is a metabolite of great importance, since it acts in the antioxidant defense system of plants and is a key substrate for detoxification and stable maintenance of reactive oxygen species (ROS) within chloroplasts, peroxisomes, mitochondria, and cell cytosol [36].

Phenolic compounds are a chemically diverse group of secondary metabolites that occur ubiquitously in plants [37]. They can be divided into several groups, including phenolic acids, flavonoids, stilbenes, and lignans, depending on their chemical structure [38]. They commonly act as chemical and physical barriers to protect plants against biotic and abiotic stress [39], provide protection against excess sunlight by absorbing high energy wavelengths [40], and they can also protect plants from various pests and diseases [41].

According to the results obtained in this work, as well as those reported in the literature, CNMs can modify the antioxidant defense system of plants through the increase of antioxidant compounds. This is because CNMs can induce slight oxidative stress in plants [16] and overexpress the genes involved in stress signaling in plants [2]. This results in the production of antioxidant compounds, such as phenolic acids and flavonoids [2], ascorbic acid [16], polyphenolic compounds [42], and others, which ultimately decrease oxidative stress and improve defense against other types of biotic or abiotic stress. However, the observed responses depend on the concentration of CNMs applied, since a hormetic response can be observed where low concentrations can induce positive effects but high concentrations induce the opposite effect, and, in addition to higher concentrations, a positive effect can be observed again. This hormetic effect is commonly observed in plants, since it is a dynamic adaptive response of complex biological systems to different stressors [43].

Antioxidant enzymes are part of the antioxidant defense system of plants and, in turn, are considered proteins. It is known that the application of CNMs, such as carbon nanotubes, have the ability to induce the production of antioxidant enzymes which results in an accumulation of proteins [25]. In addition, it has been reported that the application of nanomaterials can increase the content of aquaporins, a type of protein specializing in the transport of water located in the membranes [44]. An increase in the proline amino acid has been reported in carrot plants when graphene oxide was applied at 0.10 mg L^−1^ [32]. This is consistent with what was observed in the present study, since it was demonstrated that both CNTs and graphene induce a greater accumulation of proteins in tomato seedlings.

Antioxidant enzymes play a very important role in plants, as they protect cells from alterations induced by oxidative stress, because they neutralize reactive oxygen species [42]. Although CNMs can induce oxidative stress in plant cells [45], due to the ability they have to translocate to the different organelles of the cells [7], the result is beneficial for the plant, since it induces the generation of free radicals [46] which leads to the activation of enzymes to mitigate oxidative stress. Among these enzymes is APX which helps decrease the oxidative state of chloroplasts due to the neutralization and balance of ROS [36]. Both GPX and CAT are other major enzymes responsible for the elimination of ROS and catalyze the reduction of H_2_O_2_ to prevent cell damage [47]. The results of this work showed that the activity of different antioxidant enzymes in tomato seedlings increased due to the application of CNMs which is consistent with that reported by various authors. In a crop of beans (*Phaseolus vulgaris*), the application of graphene oxide (400 and 800 mg L^−1^) increased the activity of APX and CAT; however, at a higher dose (1600 mg L^−1^), it generated the opposite effect [48]. Rong et al. [25] showed that the application of multi-walled carbon nanotubes (2.5, 5, and 10 mg L^−1^) increased the activity of APX and CAT in a crop of beans (*P. vulgaris*) under heavy metal stress.

The PAL enzyme, although it does not have an antioxidant function directly, is important in the antioxidant defense system due to the fact of its participation in the phenylpropanoid pathway [49]. These are precursors of phenolic compounds that give the plant greater antioxidant capacity, in addition to increasing defense against pathogens, improving tolerance against biotic stress [50]. This enzyme was also stimulated by the application of CNMs, especially foliar pathways, so it can be expected that this type of nanomaterial will confer greater tolerance against pathogens to tomato seedlings.

## 4. Materials and Methods

### 4.1. Crop Growth

Tomato seeds of the variety “Pony” (Harris Moran, Davis, CA, USA) of saladette type and determined growth were used. Direct sowing was carried out, placing a tomato seed in a 1 L polystyrene container. Ten repetitions were used per treatment, considering one seedling as an experimental unit. A mixture of peat moss, perlite in a 1:1 ratio, was used as a substrate. For the seedling nutrition, Steiner solution was applied [51]. The crop was developed for 60 days from sowing.

### 4.2. Characteristics of Carbon Nanomaterials and Treatments

**Figure 7:**
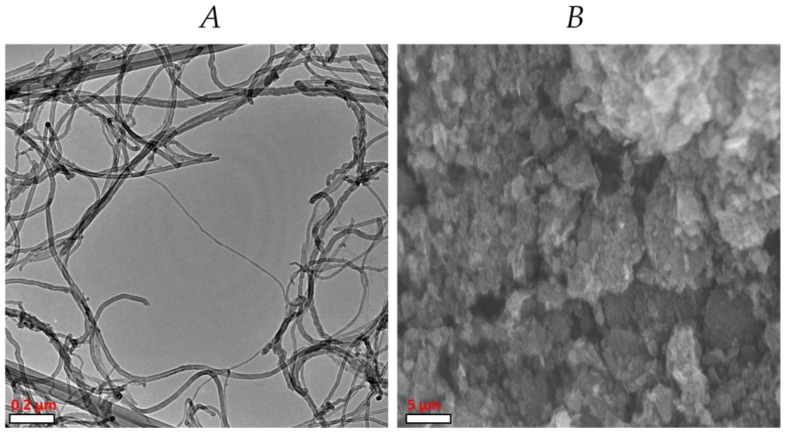
Carbon nanotubes (**A**) and graphene (**B**) obtained by scanning electron microscopy.

Two types of carbon nanomaterials were used: carbon nanotubes and graphene. The carbon nanotubes (CNTs) were multilayer with a diameter of 30–50 nm, length of 10 to 20 μm, and a purity of approximately 95% (Nanostructured & Amorphous Materials, Inc., Houston, TX, USA) (Figure 7A). The graphene (GP) used was multilayer with a diameter of 2 μm, a thickness of 8 to 12 nm, and a purity of 97% (Cheap Tubes Inc., Cambridgeport, VT, USA) (Figure 7B). The dispersion of the CNMs was determined through the Z potential, using a Z potential analyzer (ZetaCheck, ZC 0006, Microtrac, Montgomery, PA, USA), being −39.1 mV for the CNTs and −35.2 mV for the GR.

The treatments consisted of the use of CNTs and GP in different doses (10, 50, 100, 250, 500, and 1000 mg L^−1^) and applied via foliar or drench, plus a control. The treatments were applied only once, 45 days after sowing (dds), applying 1.5 mL of each solution for each seedling. In the case of the control, only distilled water was applied.

### 4.3. Sampling

After 60 dds, samples of the third fully developed young leaf were taken to perform the biochemical analyses. The fresh shoot and root biomass were quantified and dry shoot and root biomass were obtained after drying for 48 hours at 80 °C.

### 4.4. Biochemical Analysis

The content of chlorophyll was determined according to the method of Nagata and Yamashita [52]. The absorbances at 645 and 663 nm were determined and used in Equations (1) and (2) to determine the content of chlorophyll, as follows:

(1) $$\mathrm{Chl}\;a=0.999\times {\mathrm{Abs}}_{663}-0.0989\times {\;\mathrm{Abs}}_{645}$$

(2) $$\mathrm{Chl}\;b=-0.328\times {\mathrm{Abs}}_{663}+1.77\times {\;\mathrm{Abs}}_{645}$$

The total chlorophyll is the sum of Chl a and Chl b. All data are expressed as mg 100 g^−1^ dry weight (mg 100 g^−1^ DW).

Ascorbic acid (mg g^−1^ DW) was determined by the colorimetric method using 2,6 dichlorophenol, 1 g of fresh tissue, and HCl (2%) as described in Padayatt et al. [53].

Glutathione (mmoL 100 g^−1^ DW) was determined using the method of Xue et al. [54] by means of a 5,5-dithio-bis-2 nitrobenzoic acid (DTNB) reaction. A mix of 0.480 mL of the extract, 2.2 mL of sodium dibasic phosphate (Na_2_HPO_4_ at 0.32 M), and 0.32 mL of the DTNB dye (1 mM) was placed in a test tube. Then, the mix was vortexed and read on a UV-Vis spectrophotometer (UNICO Spectrophotometer Model UV2150, Dayton, NJ, USA) at 412 nm using a quartz cell.

Flavonoids (mg 100 g^−1^ DW) were determined by the method of Arvouet-Grand et al. [55]. For the extraction, 100 mg of lyophilized tissue was placed in a test tube where 10 mL of reagent grade methanol was added and shaken for 30 s until the mixture was homogenized. The mixture was filtered using No. 1 Whatman paper. For the quantification, 2 mL of the extract and 2 mL of methanolic solution of aluminum trichloride (AlCl_3_) 2% were added to a test tube and left to rest for 20 min in the dark. The reading was then taken in a UV-Vis spectrophotometer (UNICO Spectrophotometer Model UV2150, Dayton, NJ, USA) at a wavelength of 415 nm using a quartz cell.

Phenols (mg g^−1^ DW) were determined with Folin–Ciocalteu reagent as described in Cumplido-Nájera et al. [21]. The sample (0.2 g) was extracted with 1 mL of a water:acetone solution (1:1). The mixture was vortexed for 30 s. The tubes were centrifuged (UNICO Spectrophotometer Model UV2150, Dayton, NJ, USA) at 17,500× *g* for 10 min at 4 °C. In a test tube, 50 μL of the supernatant, 200 μL of the Folin–Ciocalteu reagent, 500 μL of 20% sodium carbonate (Na_2_CO_3_), and 5 mL of distilled water were added and then vortexed for 30 s. The samples were placed in a water bath at 45 °C for 30 min. Finally, the reading was taken at an absorbance of 750 nm using a plastic cell in a UV-Vis spectrophotometer (UNICO Spectrophotometer Model UV2150, Dayton, NJ, USA).

The quantification of total proteins (mg g^−1^ of DW) was performed using Bradford’s colorimetric technique [56]. In a microplate, 5 μL of the extract and 250 μL of Bradford reagent were placed in each well. They were incubated for 10 min at room temperature (26 °C) and then read at a wavelength of 630 nm on a microplate reader (Allsheng, AMR-100 model, Hangzhou, China).

The antioxidant capacity was determined using DPPH (2,2-diphenyl-1-picrylhydrazyl) radical according to Brand-Williams et al. [57]. Hydrophilic compounds were determined using phosphate buffer for extraction and, for lipophilic compounds, a hexane:acetone solution was used. The total antioxidant capacity was obtained by the sum of the hydrophilic and lipophilic compounds [58]. The antioxidant capacity was expressed as ascorbic acid equivalents (mg g^−1^ DW)

The extract used was the same as that used for total proteins and following the standard techniques. The enzymes evaluated were ascorbate peroxidase (EC 1.11.1.11) [59], glutathione peroxidase (EC 1.11.1.9) [54,60], catalase (EC 1.11.1.6) [61], and phenylalanine ammonia lyase (PAL) (EC 4.3.1.5) [62].

### 4.5. Statistical Analysis

For the evaluation of the agronomic variables, 10 repetitions per treatment were considered and, for the biochemical variables, five repetitions per treatment. A completely randomized design was used, and analysis of variance and Duncan means test (*α* = 0.05) were performed using the InfoStat software (v2018) (https://www.infostat.com.ar).

## 5. Conclusions

The application of carbon nanomaterials in both routes of application (i.e., foliar and drench) modifies the antioxidant defense system of tomato seedlings generally positively. However, for each specific antioxidant compound, the responses were different depending on the type of carbon nanomaterial used (carbon nanotubes or graphene), the route of application (foliar or drench), as well as the doses used.

Additionally, the application of CNMs increased the chlorophyll content regardless of the route of application and, especially, with the application of graphene. The content of antioxidant proteins and enzymes also increased.

The results show that the antioxidant defense system of tomato seedlings can be improved which can potentially increase tolerance against various types of biotic or abiotic stress. In addition, no toxicity was observed for any dose of CNMs used, since there were no negative effects on biomass production.

## Abbreviations

CNMs	carbon-based nanomaterials
CNT	carbon nanotubes
GP	graphene
SWCNT	single-wall carbon nanotubes
MWCNT	multi-wall carbon nanotubes
DPPH	2,2-diphenyl-1-picrylhydrazyl
TAC	total antioxidant capacity
GSH	glutathione
APX	ascorbate peroxidase
GPX	glutathione peroxidase
CAT	catalase
PAL	phenylalanine ammonium lyase
